# Tokophobia in Iranian women during the COVID-19 pandemic

**DOI:** 10.18502/ijrm.v19i11.9918

**Published:** 2021-12-13

**Authors:** Saleheh Tajalli, Asad Imani

**Affiliations:** ^1^Nursing Care Research Center (NCRC), School of Nursing and Midwifery, Iran University of Medical Sciences, Tehran, Iran.; ^2^Department of Nursing, Faculty of Nursing and Midwifery, Ilam University of Medical Sciences, Ilam, Iran.

##  Dear Editor, 

On March 11 2020, the World Health Organization declared that coronavirus disease (COVID-19) was a worldwide pandemic (1). This virus belongs to the group of beta-coronaviruses (2). At first it appeared that people aged over 60 yr had a higher risk of respiratory disabilities and death as a result of COVID-19 infection (3), but that pregnant women were not adversely affected (4). Then later, pregnant women were classified as an at-risk group; recently a report showed that COVID-19 infection during pregnancy can lead to adverse clinical consequences including maternal disease and life-threatening complications. Some mothers infected with COVID-19 have required hospitalization, intensive care, and invasive/noninvasive ventilation. Also spontaneous abortion, perinatal death, intrauterine growth restriction, preterm delivery, and admission to the NICU are possible (5). COVID-19 infection in pregnant women with severe respiratory signs and symptoms is usually accompanied by maternal and neonatal adverse health consequences: low birth weight, preterm birth, maternal mortality, and eclampsia (6, 7). Undoubtedly, pregnant women experience a worsening of signs and symptoms throughout and after pregnancy when infected with COVID-19 (8, 9). Adverse results of this specific condition are stress, anxiety, forced isolation, loneliness, and depression, which are heightened in pregnant women (10, 11). These women have additional concerns about their own and their unborn baby's health. During the COVID-19 pandemic, restrictive public health actions were implemented to reduce community transmission: quarantine implementations, border closures and travel bans, isolation and physical distancing, interaction limitation, and decreased access to social support (12, 13). Coronavirus is rapidly spreading around the world and its psychological effects are increasing gradually (14). Certainly, COVID-19, as a novel coronavirus, is responsible for overwhelming emotional and psychological changes. During this pandemic, pregnant women and other vulnerable groups are unprotected from the high levels of stress and other psychological disorders.

One of the most remarkable concerns related to pregnant women is tokophobia. This is a pathological fear of pregnancy and can lead to avoidance of childbirth (15). Recently it was concluded that women's expectations and concerns about childbirth changed during the COVID-19 worldwide pandemic (10).

In Iranian pregnant women, some health behavioral changes have been seen, according to our clinical experiences and field observations, which can lead to tokophobia, such as avoiding going to health centers to obtain maternity care services, choosing private hospitals instead of governmental hospitals for delivery (with these women assuming that fewer patients are admitted to these hospitals), and electing for a cesarean section instead of vaginal delivery (following from a fear of transmitting the virus from mother to fetus during delivery). Therefore, the Ministry of Health, as the main institution responsible for health promotion in Iranian society, should take the following strategies to reduce the incidence of tokophobia:

1. Designating special health centers for pregnant women in each province, which are separated from     COVID-19 screening centers.

2. Determining special wards in the maternal hospitals for women suspected of being infected with COVID-19.

3. Prioritizing pregnant women, as a vulnerable population, for COVID-19 screening tests and vaccination.

4. Assessing anxiety and distress in pregnant women during the COVID-19 pandemic.

5. Focusing on the cultural and/or ethnic minorities of pregnant women during the COVID-19 crisis.

6. Considering specific social media for psychological and clinical education of pregnant women and their       partners.

7. Educating, consulting, and following pregnant women and their partners using telemedicine and     telenursing due to the reduction in face-to-face contact.

8. Focusing on pregnant women as a high-risk group in vaccination programs.

##  Conflict of Interest 

Nothing to declare.
